# Quality principles of retrospective data collected through a life history calendar

**DOI:** 10.1007/s11135-022-01563-x

**Published:** 2022-10-27

**Authors:** Julie Chevallereau, André Berchtold

**Affiliations:** 1 LIVES Centre, Swiss Centre of Expertise in Life Course Research, Lausanne, Switzerland; 2 Institute of Social Sciences, University of Lausanne, Lausanne, Switzerland

**Keywords:** Retrospective data, Data collection, Life history calendar, Data quality, Self-administration accuracy

## Abstract

To assert the quality of retrospective data, most studies using tools such as life history calendars rely on comparisons with external sources. Our research aimed to integrate quality principles into a life history calendar and test their capacity to evaluate the data quality. The purpose was to avoid reliance on external data sources because of their possible unavailability. The first quality principle was the relationship between the dating accuracy of verifiable events and the data quality of the life domains of the calendar. The second was the certainty, as self-assessed by participants through color coding, that an event took place at the quarter indicated. We designed an experiment using a paper-and-pencil life history calendar that was completed by 104 university students. Our research highlighted the relevance to use the self-assessment of certainty to assert the data quality. However, we could not establish a relationship between the dating accuracy of verifiable events and the data quality of the life domains. In addition, we present a set of qualitative findings from 20 interviews conducted with study participants explaining the approaches used to complete a life calendar and the difficulties encountered.

**Supplementary Information:**

The online version contains supplementary material available at 10.1007/s11135-022-01563-x.

## Introduction

The life history calendar (LHC), also known as the event history calendar, is a tool for collecting retrospective data and is used in different research fields, such as life course studies, criminology, and sexuality (Sutton, 2010; Morselli et al., 2016a; Bay-Cheng, 2017). It consists of a kind of diary in which respondents can indicate the dates of events in their lives that belong to several predefined categories (education, employment, family life, health problems, etc.). Several studies have highlighted the benefit of using this tool to stimulate memory processes associated with the recollection of past events (Belli, 1998; Freedman et al., 1988). Indeed, the design of an LHC improves the retrieval of past events by stimulating different memory mechanisms, such as top-down, sequencing, or parallel retrieval mechanisms (Belli, 1998). While top-down and sequencing mechanisms rely on the relationships between events within the same life domain, parallel retrieval is based on the relationships between events in different fields (Belli, 1998).

Depending on the context, the calendar instruments vary in design, format, or data collection period. The administration method may also differ between studies. An interviewer can assist the participant in completing the calendar or it can be self-completed. The format of the calendar can be either paper or online. These different calendar designs reveal the current lack of standardization of this tool and a lack of theoretical basis, especially regarding the effect on recall accuracy of specific calendar features (Glasner and van der Vaart, 2009). There is thus a need for more methodological research, especially concerning data quality. Indeed, several studies have shown that calendar instruments improve data quality in comparison with more “traditional” collection tools, but few of them analyze the methodological aspects of data quality or data validation.

The issue of quality is often raised regarding retrospective data. Indeed, these data may be subject to inconsistencies, gaps, or errors. These “memory failures” can be caused by memory lapses, bias, or specific phenomena (Grémy, 2007). Some of these phenomena, such as the telescoping effect or calendar effect, affect the data reliability of dated events by participants. The telescoping effect refers to a dating discrepancy due to the perception of recent events as being older (backward telescoping) or older events as being more recent (forward telescoping) (Janssen et al., 2006). The calendar effect occurs when past events are dated more frequently close to school terms (Shum, 1998). These memory failures interfere with the accuracy of the data collected.

In the literature, researchers use different methods to check the reliability of retrospective data. Most of these methods are external validity mechanisms, as they rely on the data collected outside the calendar. Indeed, researchers compare the data collected from LHCs to concurrent reports or official records, or even by using more traditional collection tools such as questionnaires. Freedman et al. (1988) compared retrospective data collected from an LHC in 1985 to data obtained from interviews in 1980. They did not use tests of validity but highlighted evidence of data reliability (Freedman et al., 1988). Inconsistencies in the data were detected and discussed directly with the interviewer, who received intensive training. Morselli et al. (2016b) compared data obtained from an LHC to a traditional questionnaire administered two weeks later. They concluded that the performance of the two tools was almost identical, with slightly more accurate data obtained with the LHC. Other studies (e.g. Belli et al., 2001, 2007) have compared the LHC with a traditional approach using lists of questions, randomly allocating respondents to one method or the other. The results show that LHCs almost always produce better quality results than the question list approach. Some studies use official sources, such as justice records or urinalyses, to assess data accuracy (Anglin et al., 1993). Berchtold et al. (2022) tested the consistency between several events placed on a life calendar.

Most of these studies compare calendar data with similar data or other sources to corroborate the robustness of the information. However, there is a lack of studies on validation criteria integrated into the LHC. Some studies verified the accuracy of known events dated by participants to analyze memory effects, such as the telescoping effect. Huttenlocher et al. (1988) and Kurbat et al. (1998) used known dates (movies and social activities on campus) to verify the events recalled by students, with the aim of analyzing bias dating. Their results did not confirm the initial hypotheses of the dating bias theory but led to further questioning (Shum, 1998). Other studies analyzed the influence of date formats (absolute or relative time format) for dating news and assessed the telescoping effect (Janssen et al., 2006). The researchers relied on public events selected from the Amsterdam Media Questionnaire (Klomps, 2001) and the Daily News Memory Test (Meeter et al., 2005). These studies resorted to events with known dates to assess memory effects and better understand how people date events. However, none of them, as far as we know, use these methods for assessing the data quality of a calendar instrument.

The purpose of this research was to investigate mechanisms internal to life calendars that would allow for the assessment of the quality of retrospective data collected using this tool. We wanted to avoid relying on external data sources that were not always available or appropriate. The first investigated principle of the study was the relationship between the dating accuracy of verifiable events and the data quality of the life domains (place of residence, educational and professional trajectories, and vacation) of the calendar. Two categories of events with verifiable dates were defined: the first condition domain consisted of events that participants attended (for instance, in the fields of culture, politics, and sports), and the second consisted of public events they remembered, in the same fields. Indeed, there is considerable interest in the literature regarding how we remember and recall these two types of events, both in the short and long term, as well as in the relationships between them (e.g. Brown, 1990; Toglia et al., 1992). Larsen and Thompson (1995) have shown, for example, that it is easier to remember the day of the week on which an event occurred if that event is private rather than public. The second principle is based on the certainty, as self-assessed by participants through color coding, that the events took place at the time indicated on the calendar. Thus, we sought to analyze the ability of individuals to self-assess the quality of the events they had indicated on the calendar. The choice of a color code (yellow when they were sure and orange when they had doubts) was intended to quickly visualize the level of certainty.

Our first hypothesis was that participants who correctly placed events they attended or public events (depending on the test condition) completed the calendar with higher data quality. We expected to observe fewer inconsistencies or errors in the data of other life domains. The second hypothesis was that the events that participants attended were dated more accurately than public events. Indeed, personal events in which we are directly involved as participants should be better remembered. The third hypothesis concerned the degree of certainty associated with each event (being sure or doubtful) through a color code. The error rate in dating was supposed to be lower for the events highlighted in yellow (when participants were sure about the date) than for the events in orange (when they were doubtful). A large number of yellow events on a calendar should be associated with better overall data quality of the calendar.

## Methods

This study was conducted in two phases. In the first phase, participants completed an LHC on a paper sheet and a short sociodemographic questionnaire composed of three questions. In the second phase, participants who gave their consent (at the end of the first phase of the study) were interviewed a few weeks after the experiment. The protocol of this study was approved by the Ethics Committee of the Faculty of Social and Political Sciences of the University of Lausanne (number C_SSP_102021_00004).

### The first phase: calendar experiment

#### Material

The LHC was printed on an A3 paper sheet. The paper format allowed participants to view their entire calendar at a glance without having to scroll on a computer screen. In addition, developing an online tool would have required much more time and resources.

The data collection period extended from 2015 to 2021 and was represented by seven columns divided into quarters. This choice was justified by the A3 format we had chosen and by the fact that our experiment did not necessitate exploring distant memories. The calendar was also composed of four rows corresponding to four different domains: place of residence, educational and professional trajectories, vacation, and either events attended by the respondents (condition 1) or public events (condition 2). Appendix B shows the LHC of condition 1.

For our study, the first three lines (place of residence, educational and professional trajectories, and vacation) are called the “life domains”, and the last line (either events attended by the respondents [condition 1] or public events [condition 2]) is called the “condition domain.” We distinguished these two categories of domains because the condition domain (the last line) has a particular function in our research.

An instruction sheet accompanied the calendar and explained how to fill in the calendar (see Appendix C for the version of condition (1). A distinction was made between periods and events: a period lasted at least two months and was represented by a line and dots at the beginning and end of this line, while a one-time event was represented by a single dot.

Some examples were provided for each life domain. For the categories “attended events” and “public events,” four examples were selected to illustrate different fields (culture, sports, politics, and environment) and different periods of time (very recent events as well as events that happened before the data collection period), providing participants with a wide range of references. The dates of the examples were not mentioned in order not to influence the participants and avoid that they reuse these examples in their calendar.

Other instructions were also printed directly on the calendar to remind participants of the distinction between periods and events. They also specified what was expected for each line and explained certain criteria. For instance, vacations had to have lasted at least one week. This was done to filter out weekends and other very short stays that could have been extremely numerous for some participants.

#### Test conditions

In this experiment, two quality principles were tested to analyze their capacity to quantify the data quality of the calendar. For the first principle, each participant was randomly assigned to one of the following two conditions that differed only by the condition domain (the last line of the calendar):

- *Condition 1*: The participants had to provide and date a maximum of 10 events that they attended as spectators or volunteers in different areas (for instance, culture, politics, or sports).
- *Condition 2*: The participants had to provide and date a maximum of 10 public events that they remembered in different areas (for instance, culture, politics, or sports).

The instructions printed on the calendar for condition 1 specified that the participants should provide events they attended with at least 100 people present. Indeed, this threshold was a way to filter events and keep only the major ones, which were easier to verify. To increase the possibility of identifying and verifying the event, additional information (in addition to the place) was also requested from the participants. It was especially required for recurring events that took place every year (or a certain number of years), such as a music festival. For these events, without specific information such as the name of a specific singer, it would have been impossible to verify the accuracy of the date. In the examples, several types of information were provided to illustrate what we expected. For instance, for a music festival, the name of the rock band Placebo was indicated. The second category, public events, was supposed to be easily verifiable, and no additional information was requested.

For the second quality principle, all the participants of both conditions had to highlight (once the calendar was completed) all events either in yellow when they were sure about the date or in orange when they were doubtful. This way, they indicated their level of certainty about the exact time each event occurred.

#### Protocol of the experiment

The participants in the study were mainly students from the Faculty of Social and Political Sciences at the University of Lausanne. They were contacted through courses given in the fall semester of 2021, and they could register through a web platform. One hundred and four students participated in the experiment. Between 1 and 13 students attended the sessions from October 25 to November 5, 2021. At least one collaborator from the research team was present during each session.

Before the session, the instruction sheet and life event calendar were already placed on the table to make the choice of the modalities random (Appendices A to C). A blue pen was also provided to each participant. At the beginning of the session, participants were asked to fill in a consent form. The collaborator reminded the participants to read the instructions carefully and that all explanations were present in the experiment material. There was no mention of the fact that each participant did not fill in the same calendar. All participants began to complete the calendar at the same time. The time spent completing the calendar was recorded by the collaborator. This task took between 8 and 50 min.

Once the calendar was completed, the collaborator distributed the second part of the instructions (Appendix D) accompanied by a short sociodemographic questionnaire (Appendix E) and a request to agree to participate in the interview (Appendix F). Two differently colored highlighters were also loaned to each participant. These highlighters were used to indicate on the life calendar the level of certainty that an event took place during the quarter indicated. They were given after the calendar had been filled out, so as not to influence participants' recall of events. In addition, the blue pen initially given to the participants was replaced at the same time by a red pen, which allowed us to detect any changes made to the calendar after the highlighters were distributed. The time spent highlighting the events in yellow or orange was also recorded. The second task lasted between two and eight minutes. Once this last task and the questionnaire were completed, the participants left both documents on the table. They signed a certificate of compensation and received a voucher worth 25 Swiss francs.

### The second phase: semi-structured interviews

After the last session of the experiment, 25 participants who agreed to be interviewed were contacted again. Finally, 20 participants were interviewed between two and four weeks after their participation in the calendar experiment. These participants were selected over 82 who agreed to be interviewed, according to several criteria explained below.

In the contact email, we asked the participants to bring their resumes. Indeed, this was a way to verify the dates of some of the events provided on the calendar. A precise interview grid was built to collect the same information from all participants and facilitate the analysis of the results (see Appendix G for the grid corresponding to condition 1).

Face-to-face interviews were carried out from November 10 to November 24, 2021. They lasted between 16 and 50 min. This time variation was due to the different number of events indicated in each calendar and the time required to check the accuracy of these events. The interviews were recorded.

## Data analysis

We focused on the quality of the data collected from the calendars and not on the content of these calendars. We analyzed the completeness, consistency, and reliability of the data. That was the reason why the data provided by the participants were not transcribed in full into a database. Instead, we extracted the following data from each calendar:

- The number of events provided in each life domain and condition domain
- The number of modifications and errors
- The number of events of the condition domain whose dates were verifiable
- The number of events highlighted in yellow and orange

Moreover, for each event provided for the condition domain, which differed between test conditions, we searched to identify the true date of the event by using information available on the Internet and computed the difference between the true date and the one given on the calendar with a precision of plus or minus one-quarter.

These different data were compared between the two conditions of the experiment. We also considered other variables, such as sociodemographic variables (age, gender, and language) and time spent completing the calendar.

First, each variable was characterized using descriptive statistics (mean and standard deviation). Then, a Wilcoxon test was used to identify significant differences in some variables between the two test conditions, as the hypothesis of normality was not fulfilled. All statistical analyses were performed using the R statistical environment (R Core Team, 2021). Type I error was set at 5%.

## Results from the experiment

### Sample description

Table 1 presents the main sociodemographic characteristics of the participants. Women are overrepresented in the sample (82.7%), which is due in part to the overrepresentation of women among the students of the Faculty of Social and Political Sciences of the University of Lausanne. The mean age is 21.82 years, and most of the participants are native French speakers (85.6%). There are no significant differences between the two test conditions for these three variables.

**Table 1:** Main characteristics of the participants, overall and by condition

		Overall (*n* = *104*)	Condition 1 (*n* = *52*)	Condition 2 (*n* = *52*)	*p*-value
Gender	female	86	44	42	0.851
	male	13	6	7	
	others	5	2	3	
Age: mean (SD)		21.82 (2.95)	21.82 (2.70)	21.82 (3.21)	0.504
	*n*	*100*	*50*	*50*	
Mother tongue	French	89	45	44	1
	other	15	7	8	

When n is different from the grand total (*n* = *104*), it is specified under the variable

The Wilcoxon test was performed for age, since the hypothesis of normality was not fulfilled. The Chi-square test was performed for nominal variables (gender and mother tongue)

During the experiment, we recorded the time spent to complete the calendar (completion time 1) and the time spent to highlight the events in yellow or orange (completion time 2) (Table 2). On average, for the entire sample, the respondents completed the calendar in 27.09 min (SD: 7.36) and highlighted the events in 4.34 min (SD: 1.13). No significant differences were found between the two test conditions. Most of the participants (85.6%) completed an event history calendar for the first time, and again, there was no difference between the conditions.

**Table 2:** Completion time overall and by condition, and previous experience with an LHC

		Overall *(n* = *104)*	Condition 1 *(n* = *52)*	Condition 2 *(n* = *52)*	p-value
Completion time 1: mean (SD)		27.09 (7.36)	27.44 (7.94)	26.73 (6.77)	0.648
Completion time 2: mean (SD)		4.34 (1.13)	4.48 (1.18)	4.19 (1.07)	0.394
Already completed an LHC	Yes	12	7	5	0.713
	No	89	44	45	
	Do not remember	3	1	2	

The Wilcoxon test was performed for completion time variables because the hypothesis of normality was not fulfilled

The Chi-square test was performed for categorical variables (LHC already completed) but with a limited power, since less than 80% of the classes were above 5

### Data extracted from calendars

#### Counting of errors, modifications, and missing data

For each calendar, we counted the total number of events, the number of events highlighted in yellow (when the participant was sure about the date) and those in orange (when the participant was doubtful). We also examined modifications and errors. The modifications were counted when participants crossed out an event or the horizontal line representing a period. As far as errors are concerned, it should be noted first of all that it is very complicated to identify, on the basis of the life calendars alone, events that have been forgotten by the participants or, on the contrary, events that have been indicated but have in fact never taken place. Three categories of errors were then considered: nonobservance of the instructions, missing data, and overlapping of periods. There were several cases of nonobservance of the instructions. For instance, two categories of events were distinguished in the guidelines: periods (more than two months) and punctual events (less than two months). A period had to be represented by a horizontal line with a point at the beginning and end. In some calendars, there were many points on the same line for a single period (see Appendix H). This was counted as an error for nonobservance of the instructions. Only few calendars had this type of error.

In addition, we identified some missing data for the place of residence and educational trajectory. The place of residence was easy to identify, as there should have been information for every year of the calendar. For the educational trajectory, some missing information regarding mandatory school and current university education could be identified. Such gaps were observed in six calendars. Inconsistent overlaps were only identified in two calendars when two periods of educational trajectory happened at the same time, although they should happen one after another given that the participants in this study were young and all had completed an educational pathway that included compulsory schooling, high school and university.

#### Aggregated data

Table 3 presents the average number of events, modifications, and errors for each life domain of the calendar. On average, each participant provided 28.42 events (SD: 10.36). Vacations were the life domain with the highest number of events (11.71, SD: 7.62). Regarding the condition domain, the average number of events was lower than 10, as participants were required to provide a maximum of 10 events. Only four participants provided more than 10 events, with a maximum of 17, what was counted as an error. A few modifications (2.76, SD: 2.45) and errors (0.63, SD: 0.97) were identified. No significant differences between the conditions were observed for the different variables.

**Table 3:** Number of reported events, modifications, and errors, as well as yellow and orange highlighted events, for each life domain, overall and by condition

		Overall *(n* = *104)*	Condition 1 *(n* = *52)*	Condition 2 *(n* = *52)*	*p*-value
Residence	No. of events	2.79 (2.05)	2.67 (2.26)	2.9 (1.83)	0.164
	No. of modifications	0.31 (0.68)	0.31 (0.64)	0.31 (0.73)	0.833
	No. of errors	0.13 (0.34)	0.13 (0.34)	0.13 (0.34)	1.000
	No. of yellow events (sure)	2.66 (1.95)	2.52 (2.14)	2.8 (1.75)	0.076
	No. of orange events (doubtful)	0.14 (0.45)	0.17 (0.47)	0.1 (0.42)	0.230
	No. of not highlighted events	0.06 (0.5)	0	0.12 (0.7)	–
Educational and professional trajectories	No. of events	7.24 (3.30)	7.52 (3.66)	6.96 (2.90)	0.565
	No. of modifications	0.67 (1.31)	0.73 (1.39)	0.62 (1.24)	0.611
	No. of errors	0.26 (0.46)	0.29 (0.46)	0.23 (0.47)	0.406
	No. of yellow events (sure)	6.75 (3.16)	6.77 (3.46)	6.72 (2.85)	0.944
	No. of orange events (doubtful)	0.44 (1.07)	0.69 (1.28)	0.18 (0.72)	0.001***
	No. of not highlighted events	0.16 (1.19)	0	0.33 (1.68)	–
Vacations	No. of events	11.71 (7.62)	12.56 (9.21)	10.87 (5.57)	0.759
	No. of modifications	1.04 (1.41)	1.15 (1.50)	0.92 (1.31)	0.630
	No. of errors	0.01 (0.10)	0.02 (0.14)	0	0.327
	No. of yellow events (sure)	9.19 (6.75)	9.52 (8.03)	8.84 (5.14)	0.770
	No. of orange events (doubtful)	2.52 (2.26)	2.98 (2.58)	2.04 (1.78)	0.078
	No. of not highlighted events	0.19 (1.44)	0	0.38 (2.03)	–
Condition domain: attended events (condition 1)					
or public events (condition 2)	No. of events	6.64 (2.70)	6.94 (3.25)	6.38 (2.01)	0.280
	No. of modifications	0.75 (1.10)	0.9 (1.34)	0.62 (0.80)	0.408
	No. of errors	0.23 (0.58)	0.28 (0.70)	0.17 (0.43)	0.664
	No. of yellow events (sure)	4.49 (2.55)	4.73 (3.06)	4.25 (1.91)	0.506
	No. of orange events (doubtful)	2.04 (1.58)	1.94 (1.76)	2.13 (1.37)	0.244
	No. of not highlighted events	0	0	0	–
Total	No. of events	28.42 (10.36)	29.78 (12.81)	27.12 (7.17)	0.651
	No. of modifications	2.76 (2.45)	3.08 (2.63)	2.46 (2.26)	0.209
	No. of errors	0.63 (0.97)	0.72 (1.07)	0.54 (0.87)	0.522
	No. of yellow events (sure)	23.12 (9.81)	23.54 (11.83)	22.67 (7.17)	0.653
	No. of orange events (doubtful)	5.09 (3.03)	5.79 (3.32)	4.35 (2.51)	0.028 *
	No. of not highlighted events	0.41 (2.72)	0	0.83 (3.82)	–

The Wilcoxon test was performed, since the hypotheses of normality were not fulfilled

Significance level: *** p < 0.001, ** * p*< 0.01, * p < 0.05

Regarding the use of yellow and orange highlighting, events were more often highlighted in yellow (23.12 events on average, SD: 9.81) than in orange (5.09, SD: 3.03). This was particularly the case for the place of residence and educational/professional trajectories for which the average number of events highlighted in orange was very low (0.14 and 0.44, respectively). There was no significant difference between the two conditions except for the educational and professional trajectory and the grand total, where significantly more events were highlighted in orange in condition 1.

The interpretation of the significant difference between the conditions for the educational and professional events highlighted in orange is not straightforward. This difference seems to be a consequence of the specific distribution without relation to the condition of the calendar. Indeed, based on the interviews, few participants made links between the condition domain and educational and professional trajectories.

### Date verification of the condition domain

#### Average analysis

Table 4 summarizes the results of the verification analysis of dates from the condition domain. We compared the average number of events whose dates were verifiable between the two conditions. Unsurprisingly, significantly more public events could be verified than events attended by the respondents. Indeed, to verify the public events indicated in the calendars, we did not rely on the information provided by the participants, unlike the events they attended. Public events (condition 2) were easily verifiable, as they were one-off news events that could be found on the Internet. For example, an election or a sporting event such as the soccer World Cup in Russia (often indicated) can be checked. For events in which the respondents participated (condition 1), demonstrations or festivals were often indicated on the calendars. For these events, additional information (requested in the instructions) was needed to verify these events, which were sometimes cyclical or had taken place several times over a period.

**Table 4:** Verification of the dates of events either attended by the respondents (condition 1) or public events (condition 2), overall and by condition

	Overall *(n* = *104)*	Events attended (Condition 1) *(n* = *52)*	Public events (Condition 2) *(n* = *52)*	*p*-value
No. of pieces of information	–	4.2 (3.16)	–	–
No. of verifiable events	4.03 (2.61)	2.18 (1.86)	5.81 (1.9)	< 0.001***
No. of dating errors	1.31 (1.20)	1.03 (1.12)	1.52 (1.23)	0.329
No. yellow correct events	2.81 (1.80)	1.82 (1.16)	3.44 (1.86)	< 0.001***
No. of yellow incorrect events	0.61 (0.74)	0.88 (0.72)	0.51 (0.74)	0.427
No. of orange correct events	1.04 (0.85)	0.42 (0.58)	0.94 (1.07)	0.476
No. of orange incorrect events	0.76 (0.96)	0.83 (0.83)	1.16 (0.85)	0.616
Dating difference (in quarter)	0.88 (2.09)	1.16 (2.01)	0.78 (2.11)	0.126

The Wilcoxon test was performed, since the hypotheses of normality were not fulfilled. The p-values were adjusted using the Bonferroni method to account for multiple comparisons

Significance level: *p < 0.05, ** *p*< 0.01, *** *p* < 0.001

On average, 4.2 (SD: 3.16) pieces of information about the events which the participants attended were provided (Table 4). We observed that the average number of yellow correct events per calendar was significantly different between the two conditions. On average, more yellow events were correct for condition 2 than for condition 1. This observation may be because more yellow events were verified for condition 2, and most of them were correct. We must specify that some events of condition 1 could not be verified because their real dates could not be determined. However, since the cause of these missing data was independent of the data, they could be considered missing completely at random (MCAR, Little and Rubin, 1987). Therefore, their presence did not influence previous results. Finally, there was no significant difference between the two conditions with respect to the dating difference (gap between the reported quarter and the actual quarter of event occurrence).

#### Proportion analysis

Table 5 shows the proportion of events in the condition domain based on the highlighting color, verifiability, and accuracy. Overall, 68.8% of the events were highlighted in yellow (70.9% for condition 1 and 66.6% for condition 2), meaning that the participants were rather sure about the placement of events on the calendar. Of the total number of events for all the calendars, 60.5% were verified, with a significant difference between the conditions (31.4% for condition 1 and 91% for condition 2). This result is not surprising, as the information requested for condition 1 was not always present (60.5%) or sometimes not sufficiently precise to allow an accurate verification of the event. Regarding the color of the verified events, most of the yellow ones were correct (86.9% overall, 81.1% for condition 1, and 89.1% for condition 2), and most of the orange ones were incorrect (60.3% overall, 71.4% for condition 1, and 56.4% for condition 2), which indicates a fair judgment by the respondents. In both cases, the difference between the two conditions is statistically significant.

**Table 5:** The proportion of events either attended by the respondents (condition 1) or public events (condition 2), overall and by condition

	Overall *(n* = *104)*	Events attended (Condition 1) *(n* = *52)*	Public events (Condition 2) *(n* = *52)*	p-value
Total number of events	679	347	332	1.000
No. of yellow events (sure)	467	246	221	1.000
% of total no. of events	68.8%	70.9%	66.6%	
No. of orange events (doubtful)	212	101	111	1.000
% of total no. of events	31.2%	29.1%	33.4%	
No. of pieces of information	–	210	–	–
% of total no. of events		60.5%		
No. of verifiable events	411	109	302	< 0.001***
% of total no. of events	60.5%	31.4%	91%	
No. of verified yellow events	275	74	201	< 0.001***
% of verifiable events	66.9%	67.9%	66.6%	
% of yellow events	58.9%	30.1%	91%	
No. of verified orange events	136	35	101	< 0.001***
% of verifiable events	33.1%	32.1%	33.4%	
% of orange events	64.2%	34.7%	91%	
No. of correct yellow events	239	60	179	< 0.001***
% of verified yellow events	86.9%	81.1%	89.1%	
No. of incorrect yellow events	36	14	22	1.000
% of verified yellow events	13.1%	18.9%	10.9%	
No. of correct orange events	54	10	44	< 0.001***
% of verified orange events	39.7%	28.6%	43.6%	
No. of incorrect orange events	82	25	57	0.004***
% of verified orange events	60.3%	71.4%	56.4%	

The two-sample proportion tests were performed without continuity correction. The *p*-values were adjusted using the Bonferroni method to account for multiple comparisons. The *p*-values equal to 1.000 are a consequence of this adjustment

Significance level: *p < 0.05, **p < 0.01, ***p < 0.001

**Fig. 1:**
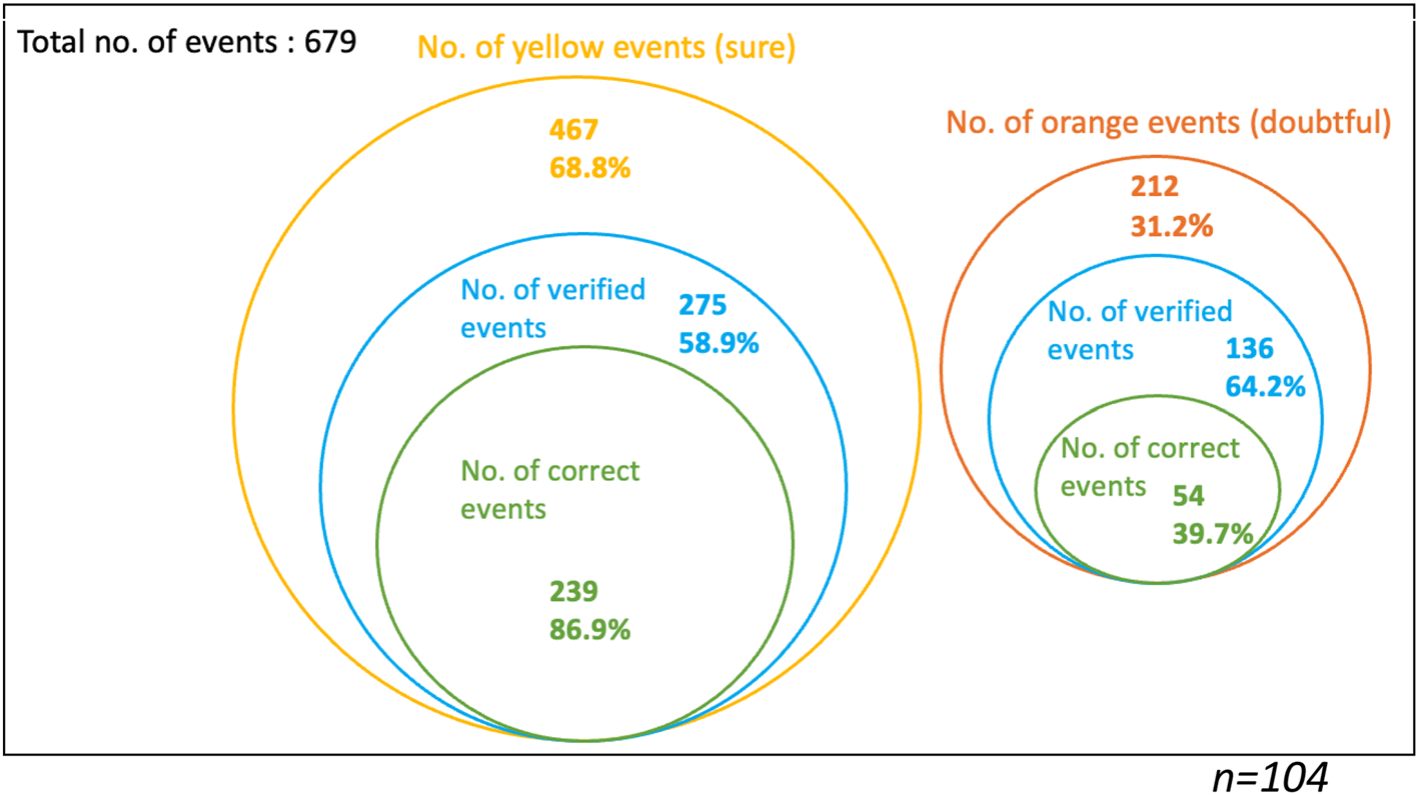
Venn diagram of the overall proportion of events both attended by the respondents (condition 1) and public events (condition 2)

**Fig. 2:**
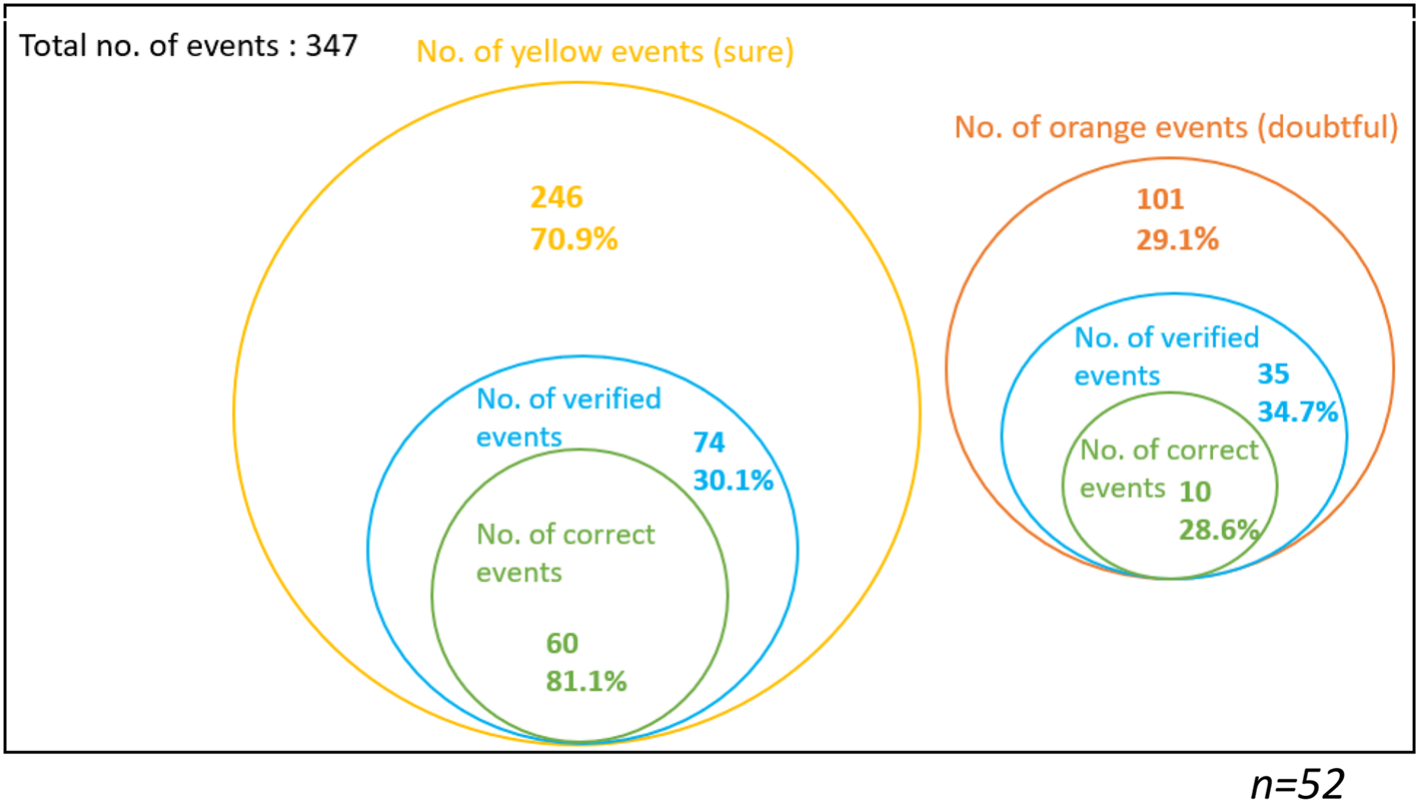
Venn diagram of the proportion of events attended by the respondents (condition 1)

**Fig. 3:**
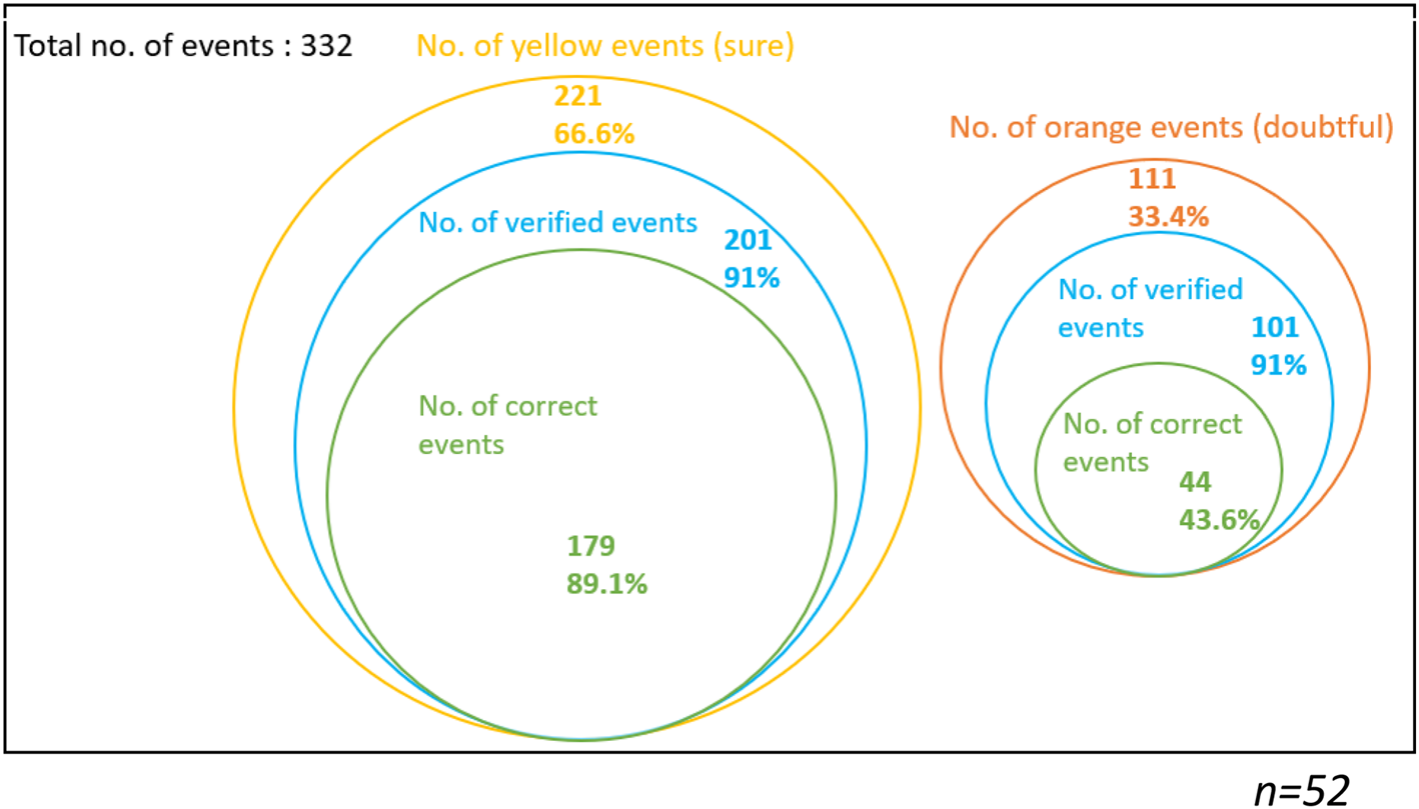
Venn diagram of the proportion of public events (condition 2)

Figures 1, 2 and 3 are another representation of Table 5, and they illustrate the proportion of yellow events (sure events) and orange events (doubtful events) over the total number of events (events attended by the participants and public events), overall and by condition. In these diagrams, we indicate the proportion of yellow events with yellow circles, the proportion of verified events with blue circles, and the proportion of correct events among the yellow verified events with green circles. The same scheme is used for the orange events.

## Results from the semi-structured interviews

### Goal of the interviews

The main objective of the qualitative part was to improve the quality of the data in the life calendar by identifying possible errors, inconsistencies, and missing data. The interviews tackled areas of life that could not be verified solely on the basis of freely available external information. They also allowed for further verification of the accuracy of some events, particularly those in modality 1, and thus to refine the comparisons between the two conditions. Finally, other information related to the way in which the life calendars were apprehended and completed could be obtained, and their use can help to improve the LHC tool as a whole with the ultimate goal of collecting the most reliable retrospective data possible.

For each life domain, we checked the dates of the events by comparing the calendars with the resumes of the respondents and sometimes by asking them to use their cell phones. In addition, we asked them about how they found this exercise, the way they completed the calendar, and how they evaluated their memory capacity.

The approach in this section is purely descriptive due to the small number of interviews conducted and the non-representative nature of the subsample used. Therefore, no statistical tests were performed.

### Selection of the participants

After the first phase of the experiment, 20 participants were interviewed between two and four weeks after the experiment. The choice of the participants interviewed was not random. We selected them based on different criteria. First, we wanted approximately half in each condition (we finally obtained 11 for condition 1 and 9 for condition 2). Then, we selected calendars with yellow events well placed and others wrongly placed, and we did the same thing for the orange events. We chose calendars with many events indicated and others with little information. We also wanted participants with completely different completion times. Some participants who underlined some periods of their educational trajectory in orange (which was rare in the sample) were also selected. We also wanted people who had already participated in a previous study with an LHC. Finally, some participants who made mistakes when completing the calendar were also selected. The purpose of this selection was to have a varied panel of calendars to conduct exploratory analyses of the relationship between certain variables and the overall level of data quality.

Table 6 presents the main sociodemographic characteristics of the participants. Women are overrepresented among the interviewees due to their overrepresentation in the sample.

**Table 6:** Main characteristics of the interviewees, overall and by condition

		Overall *(n* = *20)*	Condition 1 *(n* = *11)*	Condition 2 *(n* = *9)*
Gender	female	18	10	8
	male	1	0	1
	others	1	1	0
Age: mean (SD)		21.95 (2.42)	22.36 (2.25)	21.44 (2.65)
Mother tongue	French	17	11	6
	other	3	0	3

### Quality analysis of the calendars

The main goal of the qualitative analysis of the information collected from the calendars was to identify events wrongly positioned on the calendar (incorrect events), to count these dating errors, and to consider their highlighting color. We also tried to determine whether there was a link between the dating accuracy of the condition domain and the quality of the life domains. In addition, we explored whether other variables influenced these dating errors. The second aspect of the quality analysis dealt with the missing data that we identified and analyzed.

#### Event dating errors

Before conducting the interviews, we asked the participants to bring their resumes. This was a means of verifying the events indicated in their educational and professional trajectories. Even if a resume sometimes contained errors, we decided to use this verification method. Indeed, it was a resource that was easily accessible to students. During the interviews, they were sometimes asked to use their cell phones (with their consent) to check the dates of vacations or events they attended that we had not been able to check. It was a more certain source of verification, as the photos were dated on the phone. Thanks to these two methods of verification, 91.7% of all events indicated on the 20 calendars were verified. Among them, 17.2% were dated incorrectly.

Table 7 summarizes the data extracted from the calendars of the 20 participants. Most of the events were highlighted in yellow (78.2%), meaning that the participants were rather sure about the events indicated. Most of these yellow events were verified (97.3%), and most of them were correct (92.5%). The orange events (doubtful events) were slightly less verifiable (74.2%), as these events were often older, and there was no way to verify them. Of these, 64% were dated incorrectly. For each life domain and condition, the dating error rate of the yellow events was lower than that of the orange events. For the educational and professional trajectories, 7.9% of the verified yellow events were incorrect, compared to 61.5% of the verified orange events. For condition 1, we verified 89.5% of the events attended by the respondents. Some of the events were not verifiable because the information either was not precise enough or was not present. The interviews helped in verifying more events. Of the yellow verified events, 76.6% of the events were correct for condition 1 (events attended) and 65.7% for condition 2 (public events). Among the verified orange events, dating errors were more frequent for events attended by the participants (81%) than for public events (46.2%).

**Table 7:** Calendars of the 20 interviewees. The proportion of events by color, verifiability, and error, both overall, overall without the condition domains, and separately for each life domain and condition domain

	Overall *n* = *20*	Overall without condition domains^1^ *n* = *20*	Condition 1^2^ *n = 11*	Condition 2^2^ *n* = *9*	Residence *n* = *20*	Education *n* = *20*	Holidays *n* = *20*	Events they attended *n* = *11*	Public events* n* = *9*
Total number of events	565	424	336	229	53	163	208	76	65
No. of yellow events (sure)	441	356	258	183	49	141	166	47	38
% of total no. of events	78.1%	84%	76.8%	79.9%	92.5%	86.5%	79.8%	61.8%	58.4%
No. of orange events (doubtful)	120	64	74	46	4	18	42	29	27
% of total no. of events	21.2%	15.1%	22%	20.1%	7.5%	11%	20.2%	38.2%	41.5%
No. of verifiable events	518	389	308	210	52	153	184	68	61
% of total no. of events	91.7%	91.7%	91.7%	91.7%	98.1%	92.8%	88.5%	89.5%	93.8%
No. of verified yellow events	429	347	256	173	49	140	158	47	35
% of verifiable events	82.8%	89.2%	83.1%	82.4%	94.2%	91.5%	85.9%	69.1%	57.4%
% of yellow events	97.3%	97.5%	99.2%	94.5%	100%	99.3%	95.2%	100%	92.1%
No. of verified orange events	89	42	52	37	3	13	26	21	26
% of verifiable events	17.2%	10.8%	16.9%	17.6%	5.8%	8.5%	14.1%	30.9%	42.6%
% of orange events	74.2%	65.6%	74.3%	80.4%	75%	72.2%	61.90%	72.4%	96.3%
No. of incorrect events	89	44	56	33	3	19	23	28	17
% of verified events	17.2%	11.3%	18.2%	15.7%	5.8%	12.4%	12.5%	41,2%	29.3%
No. of correct yellow events	397	331	237	160	48	129	154	36	23
% of verified yellow events	92.5%	95.4%	92.6%	92.5%	98%	92.1%	97.5%	76.6%	65.7%
No. of incorrect yellow events	32	16	19	13	1	11	4	11	5
% of verified yellow events	7.5%	4.6%	7.4%	7.5%	2%	7.9%	2.5%	23.4%	14.3%
No. of correct orange events	32	14	15	17	1	5	8	4	14
% of verified orange events	36%	33.3%	28.8%	45.9%	33.3%	38.5%	30.8%	19%	53.8%
No. of incorrect orange events	57	28	37	20	2	8	18	17	12
% of verified orange events	64%	66.7%	71.2%	54.1	66.7%	61.5%	69.2%	81%	46.2%

^1^ “Without the condition domain” means that we only consider the three life domains (residence, educational and professional trajectories, and vacation)

^2^ Condition 1 and condition 2 include the life domains and the conditions domain (events attended by the respondents for condition 1 and public events for condition 2)

Of the 20 calendars, seven had no incorrect events identified for the life domains (place of residence, educational and professional trajectories, and vacation), and four had one error. Only one calendar had more than six errors (11 errors), as the participant shifted everything by one year on her calendar. By including the condition domains, the number of dating errors increased. There were no more calendars without errors, and only one had one error. The total number of dating errors doubled from 45 to 90 when the dating errors of the condition domains were included.

As we tried to identify a link between the quality of the condition domain and the three life domains, we conducted an exploratory graphical analysis. By plotting the number of identified dating errors of the condition domain against the number of errors in the rest of the calendar, we did not identify a particular pattern. Based on this graphical analysis, we could not establish a relationship between these two variables for any of the conditions.

We also investigated other variables that could have influenced the number of incorrect events. These variables were completion time, total number of events, and missing data. Indeed, we examined whether spending more time to complete the calendar would result in a better-quality calendar. We also examined whether the number of events reported on the calendar was related to better overall quality, as more connections could be made between the events, or, on the contrary, whether it increased the chance of making mistakes. In addition, we investigated whether calendars with more errors had more missing data. To explore these aspects and possible relationships, we constructed several plots. We did not observe any specific patterns in the graphics. Therefore, we cannot conclude that there is a relationship between the number of errors (with and without the condition domains) and the number of events, the completion time of the calendar, and the number of missing data and errors.

#### Missing data

During the interviews, we also attempted to identify missing data. Among the 20 calendars, six had no missing data and five had just one missing datum. Most of the missing data were related to the educational and professional trajectories. For this category, there were a total of 28 missing pieces of data, with two participants having respectively 8 and 5 missing pieces of data. These data concerned very short professional experiences. The person with five pieces of missing data thought that the professional trajectory concerned only people whose main activity was working. The other person completely forgot them. According to her, these professional experiences were not remarkable. For the vacations, among the 20 calendars, between six and nine vacation places were not mentioned (some participants were not sure that three vacation events occurred during the period of the calendar), due to either forgetfulness, inability to place them, or doubt about the destination.

To explore the relationship between some variables (time completion 1 and number of events) and the number of missing data, we realized some graphics. Based on our exploratory analysis, we did not observe a relationship between these two variables and the missing data. It seems that there are no more missing data on calendars with less information and with a shorter filling time.

### Analysis of the interviews

#### Difficulty of the task

The perceived level of difficulty varied among interviewees and across the domains of the calendar, but several points could be made. Most respondents found it easy to fill in the place of residence, perhaps because on average the number of reportable events in this domain was found to be significantly lower than in other life domains. The educational and professional trajectories were, in general, also easy to fill in, except for a few participants who had an “atypical” trajectory, with many short professional experiences more complicated to date. The ease of completing this life domain can be explained by the habit, as students, of updating their resumes. The educational trajectory is also a period with well-defined stages that might be easier to remember for young respondents. For the vacations, the participants were more divided. For some, it was easy to remember and date vacations, in contrast to others for whom it was very complicated, as sometimes they were not sure about the date or whether the vacation lasted at least one week (a criterion in the instruction). For the majority of respondents, the most recent vacations were easier to remember and were the most memorable ones (far away, exotic trips that required preparation). For most participants, the condition domain (either attended events or public events) was complicated to complete both in finding the events and in dating them.

#### Memory capacity

Most interviewees estimated that they had a rather good memory for remembering the events (what happened or where), but not so good memory for dating these events precisely. Some of them had a hard time remembering the events, and the events were even harder to date. On the contrary, others declared that they had a very good memory for both remembering events and dating them. The profiles of the interviewees were thus diversified with regard to this variable. Some stated that emotionally significant events were easier to remember, as well as events they attended or were involved in. For others, one-time events were more difficult to remember and date. According to some respondents, it was difficult to explain why they remembered certain events, especially when these events were not necessarily striking.

#### Filling methods

Among the participants, different filling methods were used. The two main methods used equally by the participants were chronological and ante-chronological methods. Generally, they used the same method for the place of residence and educational and professional trajectories. For the educational and professional trajectories, almost all the interviewees started with the school period and then the professional path. This can be explained by the importance of school and university for students. Only one interviewee completed both in parallel. For the holidays, the filling method was often less linear, with the more recent holidays and/or the most striking ones indicated first. For the condition domains, most of the interviewees found examples of the instruction very helpful in finding events. For public events, the respondents often relied on cyclical events with time markers such as the Olympics and elections, but most of them indicated events that affected them more particularly.

To complete the calendar, another approach was to establish links between the life domains and use, for instance, the place of residence or the educational trajectory as a landmark to date holidays, attended events, or public events. An example of the links made between the life domains was an interviewee who made an error and shifted the events for every domain on the calendar. This reveals the connections she made between the domains. However, other respondents also declared that they did not establish any links between the life domains.

We also evoked nonobservance of the instructions for completing the calendar, such as a line broken into several subperiods for a single event (instead of a single line that crosses the years). Two participants who cut the period of the same line for each year explained that they were afraid of incorrectly filling in the calendar and that they were reasoning by year and not in continuity. An example would have helped them to better show the expectations for filling in the calendar and the possibility of crossing the years with a single line. Another point was the absence of information justified by an oversight or inability to provide precise information on the event.

#### Reactions to and explanations for dating errors and missing data

The interviewees reacted differently to the misdating of events. Some were surprised not only by the events highlighted in yellow but also by some events in orange. "*That's funny that it was this late, I really thought it was in 2018*" (female, 22 years old). "*I really thought it was during my 2nd year [bachelor] that I started this experience*" (female, 24 years old). "*Is it serious? I'm shocked. I highlighted in yellow I was sure, but in retrospect it's too short for two campaigns [for federal votes]*" (male, 27 years old)*.* On the contrary, other participants were not surprised, as they really had doubts about these events. This was mainly related to older events. The errors were sometimes interpreted by the interviewees as a calculation error linked to the quarter or as an error of attention. Some interviewees were also disturbed by professional experiences that occurred simultaneously or by interruptions in their professional trajectory. One participant even realized her dating errors during the experiment when she received the highlighter and that she had shifted everything by one year.

Regarding the missing data, the interviewees stated two main reasons. The first reason was forgetfulness. They did not remember these events during the experiment. “*I have the impression that there is the impact that the things have in my life and that [these events] I really forgot it because these events did not mark me, then their dates are harder to remember*” (non-binary, 24 years old). The second reason was the fear of being wrong. When they could not situate these events in time, they preferred not to indicate them on the calendar. “*I really didn't remember what year I did this, and I thought I didn't want to be wrong*” (female, 24 years old). In addition, some participants did not indicate some professional experiences, not because they did not remember them but because they considered them as unimportant or irrelevant (because they were either very short or insignificant). “*It was a very short experience and for me it was not important*” (female, 22 years old).

#### Comparison with a previous study (2020)

Three interviewees had already taken part in a similar study carried out in 2020 (Berchtold et al., 2021) with another calendar design. The format was vertical with a longer period (10 years) and with an additional life domain (associative and cultural commitment). We showed them the calendar to remind them of its format and asked them to compare the two calendar designs. They found the calendar of the current study (horizontal design) much easier to complete than the previous one (vertical design). They said that this format looked like a calendar, which made it easier to complete. “*It allows you to have a visual overview*” (female, 24 years old). “*Here it's as if it were a succession of things (1st study) and here it's quite clear that it's a superposition (2nd study) and that the things passed at the same time and it's easier to fill in something like that*” (male, 27 years old). An interviewee added that it was also very helpful to have the educational and professional trajectories together and not separated, as in the previous study, because these events were very connected.

#### Other key points

Concerning the format and division of each year into quarters, some interviewees found it complicated to place the events, especially for academic years (as the reference period was in semesters) and vacations. “*It made me confused for the vacations because June was cut off from July”* (female, 24 years old)*.* Some would have preferred the format to be in months, but they realized that the calendar would be much longer and more precise and, thus, harder to fill in. On the contrary, for others, the division into quarters was helpful, as it also referred to the seasons. “*It seemed complicated at first when I saw, is it going to overlap? And in fact, I think it's fine and it's quite relevant for the school and academic pathway, we know that it starts in September and ends in June, so it helps us visualize*” (male, 27 years old).

Half of the interviewees mentioned the COVID-19 pandemic (and the partial lockdown) as a temporal reference to situate personal events. It became a real reference timeline (before, during, or after the COVID-19 outbreak). For one participant, COVID-19 had a “blackout” effect, in her own words, as she forgot the events that had taken place before. Another participant shifted everything by one year, as if 2020 had never happened.

Finally, some of the interviewees found this exercise interesting, stimulating, and fun. “*It is funny to see my life on a paper sheet like that”* (female, 26 years old). However, some found it tiring. “*I was tired at the end, but it was an interesting exercise, more stimulating than most of the experiments I've participated in.*” (female, 26 years old). Others realized, for instance, that they often traveled. “*By putting everything on paper I realized that I was going on vacation quite a bit*” (female, 22 years old).

## Discussion

Our results invalidate our first two hypotheses but confirm the third. Indeed, we did not find a relationship between the dating accuracy of the condition domains (neither events attended by the respondents nor public events) and the quality of the life domains (place of residence, educational and professional trajectories, and vacation). Moreover, our results refute that participants made fewer errors in dating events they attended (condition 1) than public events (condition 2). Greater involvement in an event does not increase the ability to remember and date it. This is not consistent with the findings of Larsen and Thompson (1995), but this may be due to differences in experimental design between the two studies. The experiments carried out by Larsen and Thompson focused on recent events (from one week to nine months ago), and personal events were better remembered compared to the public events because of their intertwining with what they call the “personal week schema”. In our study, as the period considered spanned several years, the ability to recall may have been influenced differently. However more verifiable events for condition 1 could inflect this result. On the other hand, our results confirmed the third hypothesis. We observed an overall error rate of sure events (highlighted in yellow) lower than that of doubtful events (highlighted in orange) both for the condition domain of the whole sample and for the life domains of the participants’ sample. The more the events indicated in yellow on a calendar, the better the quality of the calendar. This can be related to recent research on the notion of certainty during introspection processes (Sparby et al., 2020) or on the impact that a possible repetition of events in the future might have on the perception of those same events in the past (Calvillo et al., 2019). We concluded that the participants had a good capacity to self-assess their certainty that an event took place on a specific date. Thus, this approach could be systematized for studies using LHCs. The same protocol should be followed: first, the participants complete the calendar, and then, once completed, the highlighters and instructions are distributed to them in order not to influence them. In the case of online calendars, a virtual highlighter could be developed, or participants could indicate their degree of certainty through a pop-up window associated with each event.

Based on our verification analysis of the interviews, we observed overall good quality of the three life domains. This can be explained by several factors. The place of residence can be considered an essential and easy way to remember information in people’s lives, with most often only one or a few places to be indicated. Moreover, there is continuity between the places, since leaving one place coincides with the arrival in a new place. Regarding the educational and professional trajectories, our sample comprised students who may be used to often see and update their resume. Another factor that can explain the good quality of the data is the collection period, which extends over only seven years. A longer collection period might have decreased the overall quality of the data.

Regarding the calendar design and instructions, most participants complied with the instructions and filling expectations. In addition, during the interviews, the participants mostly declared that the calendar and instructions were clear. Only few participants made filling errors, such as those shown in Appendix H. To avoid such errors and improve the overall quality of the calendar, an example of an entire life domain should be completed and given to the participants. For some of them, this would have helped to show the filling expectations of this collection tool.

We identified missing data only on the calendars of the twenty interviewees. This helped us identify some points that could help participants complete the calendar and avoid missing information. We should have been more specific about certain instructions. For work experience, it would have been useful to mention that every job, even student or summer job, was expected. For the events attended by the respondents, we could have explicitly asked the participants about information that would allow us to verify these events.

Regarding the format, the horizontal design was preferred by the participants who also took part in a previous study over the vertical design. Indeed, it helped visualize all life domains globally and establish links between them.

### Limitations

The first limitation was the small sample size which limited the statistical power of our statistical analyses. Moreover, our sample was very homogeneous in terms of age, gender (mainly females) and educational level. A study with a sample of people of more varied ages and more balanced between men and women could thus make it possible to obtain results more representative of the population. The subsample used for the interviews was even smaller, but the main goal here was to obtain qualitative rather than quantitative results. However, it would have been interesting, with more available resources, to interview all participants who agreed to be interviewed.

To better differentiate the two conditions of the experiment, we should have specified in condition 2 (public events) that only events in which the respondents did not take part themselves should be mentioned. This oversight increased the intersection between the two types of events (public and private), but despite this, the vast majority of the events mentioned in condition 2 (presidential elections in the United States, terrorist attacks in France, etc.) corresponded well to what had been expected. It should also be noted that, given the obligation to cite only events that included at least 100 people in condition 1, (an obligation that was necessary to be able to evaluate the accuracy of these events externally), most of these events can also be considered as public events. In spite of this, the study of the collected data shows that the events mentioned in the two conditions are for the most part clearly differentiated.

From the outset, there were also some known limitations concerning the first modality of the condition domain (events attended by the respondents). Indeed, without precise information, it was not possible to verify the dates of these events. It was not specified in the instructions that we would be looking for the verification of their dates. Therefore, it was necessary for the participants to understand the type of information that was expected. This was sometimes the case, but not always, which limited comparison with the second condition. There was also the risk that cyclical events would be indicated, such as music festivals, as a similar example had been given. For instance, without precise information about a group, it was impossible to verify whether the event was placed in the right year. This introduced random noise into the data, limiting the ability to make comparisons.

Another limitation is the subsample of people interviewed in the second part of the study. We chose to focus on the diversity of life calendars completed by the participants and to manually select the calendars that we felt were most useful for our study. In retrospect, this may be considered an error and it would certainly have been preferable to make a much more random selection, for example based on quotas. This would have made the comparison between the two modalities of the experiment more meaningful.

It should also be recognized that the reliability of resumes used as a means of verifying educational and professional events may not have always been perfect. In fact, they were a resource that was easily accessible to students, but they could contain errors. During the interviews, some respondents identified errors in their resumes, but on the calendar, the events were placed correctly.

During data analysis, we attempted to fit linear models with two variables of interest: the number of correct yellow events and the percentage of correct yellow events over the verified yellow events. The purpose was to identify the covariates that may influence these dependent variables. Unfortunately, we did not find any relevant models that could be included in our analysis.

### Further research

Further methodological studies on the quality of data collected through an LHC are interesting to conduct. Self-assessment of certainty by participants would be relevant to use with a larger sample. Furthermore, more interviews should be conducted to verify the quality of the calendars with the respondents. This would provide more robust results and refine the analysis of variables that might influence data quality. In addition, similar studies should be conducted on other life domains, particularly on personal or sensitive domains. This would allow to determine whether the self-assessment principle is still relevant for these domains.

Further research should investigate the relationship between the dating accuracy of verifiable events and the data quality of life domains. A larger sample and more interviews would allow further investigation of this quality principle. To obtain more data for events attended by the respondents, more explanations about the expectation of verifiability of the events should be added to the instructions. More generally, the guidelines to complete the calendar must be as exhaustive as possible, especially when the calendar is self-completed, without the help of an interviewer.

To summarize, this study has shown that people completing a life calendar are quite aware of their own limitations in terms of correctly dating events and that it is possible to use this to obtain a general idea of the quality of the data collected. However, this is not sufficient to demonstrate that all the data in a life calendar are correct or not. It is then necessary to combine several modes of validation.

## Supplementary Information

Below is the link to the electronic supplementary material.

Supplementary file1 (DOCX 671 KB)
