# Supplementary material for: Quality principles of retrospective data collected through a life history calendar
Source: Qual Quant. 2022 Oct 27;57(5):4229–54. doi: 10.1007/s11135-022-01563-x (PMC9612623; doi:10.1007/s11135-022-01563-x)

# Appendices

### Appendix A: Presentation of the study

**Process of recalling memories**

Hello,

Thank you for agreeing to participate in this study on the processes of remembering past experiences. This study is anonymous, and it will never be possible to link your answers to yourself. You also have the right to discontinue participation in the study at any time without prejudice.

The study consists of two phases. First, you will be asked to complete a "life calendar" type questionnaire, followed by a traditional questionnaire. You will receive the second questionnaire when you return the completed life calendar. Detailed information on how to fill out these questionnaires can be found below, as well as directly on the questionnaires.

As a token of appreciation for your participation, you will receive a Manor voucher worth 25 francs after completing both questionnaires. If you have any questions about this study, you can contact the person in charge, Professor [Author]

Thank you very much for your participation!

**Note: Since this experiment will be conducted with other students over the next few weeks, we ask you not to talk about the content of the two questionnaires to other people, so as not to distort the experiment. Thank you!**

Professor [Author]

### Appendix B: Life history calendar: condition 1

### Appendix C: Instructions for condition 1

Please read the instructions below carefully **before** you start filling out the life calendar.

You are going to fill in a **life calendar**, a sort of diary in which you will be able to note different events that have happened to you **since 2015**. Each column corresponds to a year and each sub-column to a quarter. The rows correspond to different categories of events.

Follow carefully the **2 steps** below to fill in your life calendar:

**Step** ①: In the "Age" row, indicate for each year your age in the quarter that corresponds to your birthday.

**Step** ②: Fill in each line of the calendar, distinguishing between periods and one-time events.

| - For periods: if the event lasted **at least 2 months**:   Indicate with a horizontal line its period beginning and ending with a period, as follows:  *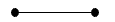Example for the place of residence (1st line of the calendar):*  Lausanne   - For one-off events: if the event lasted **less than 2 months**:   Indicate only one point in the calendar, as follows:  *Example for vacations (3rd line):*  Portugal Paris  *Example for an event in which you participated (4th line):*    Climate strike demonstration, Lausanne International film festival and forum on human rights, Geneva  Info : Presence of Greta Thunberg Info : Film Return to Homs  Youth Olympic Games, Lausanne Paleo Festival, Nyon  Info : Final of the Japanese women's field hockey team Info : Concert of Placebo |
| --- |

If **several events** took place **at the same time** in a field, indicate them, **one under the other**:

*
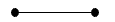
Example for academic and/or professional trajectories (2nd line):*

Bachelor, University of Lausanne

*
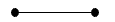
*

Seller, Coop, Lausanne

### Appendix D: Further instructions for condition 1

**Last step**: For all the events and periods you have just written in your life calendar, indicate **your level of certainty that the event took place in the quarter indicated by highlighting the event**:

| - With the **yellow highlighter** if you are **sure** that **the event took place in that quarter**.   *Example:*  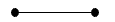  Climate strike demonstration, Lausanne Bachelor, University of Lausanne  Info: Presence of Greta Thunberg   - With the **orange highlighter** if you are in **doubt** that **the event took place in that quarter**.   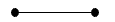*Example:*  Paleo Festival, Nyon Seller, Coop, Lausanne  Info: Concert of Placebo |
| --- |

### Appendix E. Sociodemographic questionnaire

This short questionnaire, translated from the original French version, was similar for all the participants. We will now ask you some sociodemographic questions. For each question, please check only one answer.

Q1. **What is your gender?**

€ *Female* € *Male* € *Other*

Q2. **Are you a native French speaker?**

€ *Yes* € *No*

Q3. **Have you ever had to fill out a "life calendar" in the past?**

€ *Yes* € *No* € *I do not remember*

### Appendix F. Request for consent to participate in an interview

You can still contribute to the experiment by participating in an interview lasting approximately 30 minutes. This interview will take place in person or online and will focus on the life calendar you have just completed.

At the end of the interview, you will receive a Manor voucher worth 15 francs to thank you once again for your participation!

Do you agree to be contacted again to participate in an interview?

€ I agree to be contacted for an interview. € I do not wish to participate in an interview.

If you agree to participate in an interview, please fill in your contact information.

Full name: ..................................

E-mail address : ..............................

### Appendix G: Interview grid for condition 1

Part 1:

Q1. **In general, how did you find this exercise? Was it easy, somewhat easy, somewhat difficult, or difficult to complete the calendar?**

Q2. **Which line on the calendar was the easiest to complete?**

Q3. **Which line was the most difficult?**

Part 2:

Q4. **In general, how would you rate your ability to remember events that have happened to you: poor, somewhat poor, somewhat good, good?**

Part 3:

We are now going to go back to the last line of the calendar (the events in which you participated). You indicated events.

Q6. **How did you go about filling in this last line? What strategies did you use?**

Q7. **Did you use the other lines of the calendar to fill in this last line?**

**If so, which one(s)?**

Q8: **And how did you fill in the other lines?**

On the events indicated. are not verifiable.

Q9: **Do you have the possibility of verifying the date of this event? If not, can you give me additional information about this event?**

| **Event number** | **Color of the event (yellow or orange)** | **Verified and if correct or incorrect / Unverified** |
| --- | --- | --- |
|  |  |  |

Part 4:

We are now going to look line by line at the different events you have written in the calendar, and you are going to tell me if you identify any events/periods that are either misplaced or missing.

###### Table 8: Summary table

|  | **No. of events** | **No. of yellow** | **No. of orange** | **No. of verified yellow** | **No. of verified orange** |
| --- | --- | --- | --- | --- | --- |
| Residence |  |  |  |  |  |
| Educational and professional trajectories |  |  |  |  |  |
| Vacation |  |  |  |  |  |

###### Table 9: Table of missing data

|  | **Missing data number** | **Type of missing data**  (Event or period) | **Detail** | **Reason** |
| --- | --- | --- | --- | --- |
| Residence |  |  |  |  |
| Educational and professional trajectories |  |  |  |  |
| Vacation |  |  |  |  |

###### Table 10: Table of dating errors

|  | **Error number** | **Type of error**  (Event, or beginning/end of a period) | **Color of the event (yellow or orange)** | **Detail** |
| --- | --- | --- | --- | --- |
| Residence |  |  |  |  |
| Educational and professional trajectories |  |  |  |  |
| Vacation |  |  |  |  |

### Appendix H: Extracts from calendars

###### Figure 11: Examples of nonobservance of the instructions for the place of residence


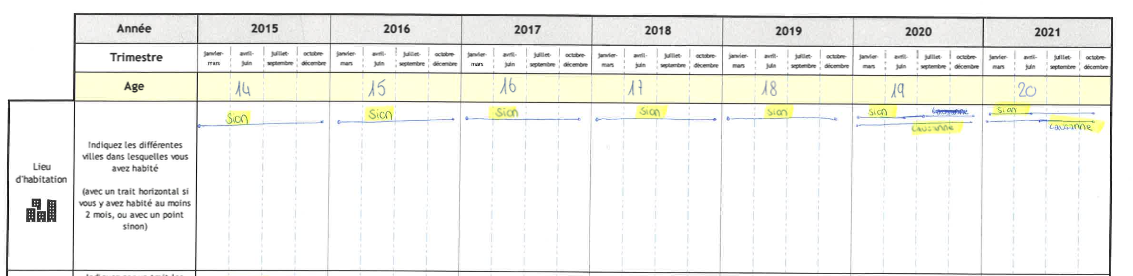


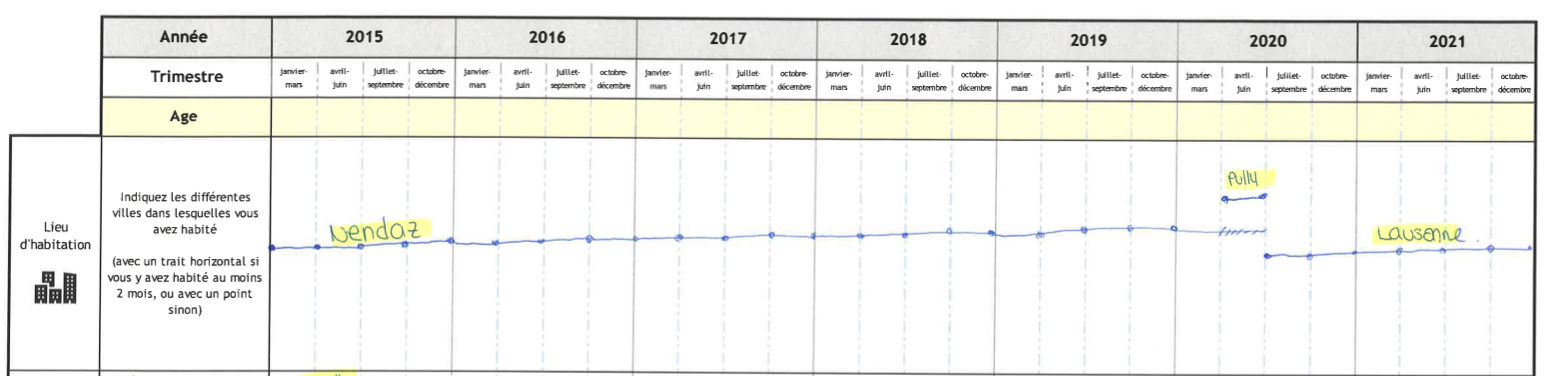

Supplement: Supplementary file 1 — Supplementary file1 (DOCX 671 KB) [file 11135_2022_1563_MOESM1_ESM.docx]
